# In Vivo Immunogenic Response to Allogeneic Mesenchymal Stem Cells and the Role of Preactivated Mesenchymal Stem Cells Cotransplanted with Allogeneic Islets

**DOI:** 10.1155/2017/9824698

**Published:** 2017-05-03

**Authors:** Régis Linhares Oliveira, Pedro Cesar Chagastelles, Patrícia Sesterheim, Patricia Pranke

**Affiliations:** ^1^Stem Cell Laboratory, Fundamental Health Science Institute, Universidade Federal Rio Grande do Sul, Porto Alegre, RS, Brazil; ^2^Post Graduate Program in Physiology, Universidade Federal Rio Grande do Sul, Porto Alegre, RS, Brazil; ^3^Fundação Estadual de Produção e Pesquisa em Saúde, Porto Alegre, RS, Brazil; ^4^Hematology and Stem Cell Laboratory, Faculty of Pharmacy, Universidade Federal Rio Grande do Sul, Porto Alegre, RS, Brazil; ^5^Stem Cell Research Institute, Porto Alegre, RS, Brazil

## Abstract

Mesenchymal stem cells (MSCs) are multipotent cells capable of differentiating into cells from the mesenchymal lineage. The hypoimmunogenic characteristic of MSCs has encouraged studies using allogeneic MSCs for the treatment of autoimmune diseases and inflammatory conditions. Promising preclinical results and the safety of allogeneic MSC transplantation have created the possibility of “off-the-shelf” clinical application of allogeneic cells. This study has aimed to evaluate the survival of untreated and IFN-*γ*- and TNF-*α*-treated (preactivated) allogeneic MSCs transplanted under the kidney capsule of immunocompetent mice together with the role of preactivated MSCs after cotransplantation with allogeneic islets. The preactivation of MSCs upregulated the gene expression of anti-inflammatory molecules and also enhanced their immunomodulatory capacity in vitro. In vivo, allogeneic MSCs provoked an immunogenic response, with the infiltration of inflammatory cells at the transplant site and full graft rejection in both the untreated and preactivated groups. Allogeneic islets cotransplanted with preactivated MSCs prolonged graft survival for about 6 days, compared with islet alone. The present results corroborate the hypothesis that allogeneic MSCs are not immune-privileged and that after playing their therapeutic role they are rejected. Strategies that reduce allogeneic MSC immunogenicity can potentially prolong their in vivo persistence and improve the therapeutic effects.

## 1. Introduction

Mesenchymal stem cells (MSCs) are multipotent cells with the capacity of proliferation and differentiation into osteoblasts, adipocytes, and chondrocytes [1]. Their ability of self-renewal and high proliferation capacity make them good candidates for cell therapy and tissue regeneration [2]. There are many therapeutic effects of MSCs; however, these effects are attributed to the secretion of paracrine factors [3–5]. The application of MSCs has mainly exploited their immunomodulatory properties, with the aim of controlling inflammatory processes of acute injuries, autoimmune diseases, or transplanted tissue or organ rejection [6–10]. The anti-inflammatory potential is mediated by suppressing the proliferation or function of T lymphocytes [4, 11], natural killer cells [12], and antigen-presenting cells (APCs) [13]. Their hypoimmunogenic characteristic and the safety of allogeneic MSC transplantation in humans have created the possibility of “off-the-shelf” application. The clinical application of autologous MSCs has limitations, which include time for in vitro expansion precluding their use in acute lesions, the high variability in the secretory pattern, and immunomodulatory properties from donor to donor [9]. As a result, more than 50% of clinical trials use allogeneic MSCs [5, 8]. Despite that, further studies have reported an immune cell response against allogeneic MSCs that vary from antidonor T cell response [14–17], production of antidonor antibodies [15–17], accelerated organ rejection [15, 17], or cell rejection after rechallenge [14, 16] while others reported long-term survival [18]. Efforts have been made to improve MSC immunosupressive and tolerogenic potentials and prolong MSC engraftment [19]. One of the approaches is the preactivation or licensing of MSCs with cytokines, such as IFN-*γ*, IL-1*β*, and TNF-*α* [19, 20], creating a proinflammatory environment to stimulate MSC immunosuppressive properties [20]. IFN-*γ*-stimulated MSCs shave increased immunosuppressive capabilities [21] and are more efficient in the prevention of colitis [22], graft-versus-host disease (GVHD) [23, 24], and autoimmune encephalomyelitis [25] and in wound healing [26] compared to untreated cells.

To better understand the biology of MSCs after transplantation, this study has aimed to evaluate the survival of untreated and preactivated allogeneic MSCs transplanted into the subcapsular space of kidneys of immunocompetent mice and to study the role of preactivated MSCs after cotransplantation with allogeneic islets in mice.

## 2. Methods

### 2.1. Animals

Mice from C57Bl/6 and BALB/c lineages were used in in vitro experiments and as receptors in the transplants. The C57/BL6-TgN(beta-act-EGFP) transgenic mice served as donors for the isolation of the MSCs. The animals were housed under standard conditions in an environment with controlled light and temperature. Water and standard mouse diet were offered ad libitum. The animal experiments followed the ethical principles of animal experimentation of the Conselho Nacional de Controle de Experimentação Animal. The project was approved by a local Ethics Committee.

### 2.2. Isolation of MSCs

Adipose tissue was collected aseptically from the animals and minced into small pieces. The fragments were digested with 1 mg/mL of collagenase type I (Gibco, Carlsbad, CA, USA) and diluted into serum-free Dulbecco's modified Eagle's medium (DMEM) for 45 min. The enzymatic activity was interrupted using DMEM supplemented with 10% fetal bovine serum (Cultilab, Brazil), 0.1 mg/mL streptomycin, and 100 U/mL penicillin. After centrifugation at 300 ×g, each pellet was resuspended in a supplemented medium and incubated in a humidified chamber at 37°C and 5% CO_2_. The cells were split using 0.05% trypsin-EDTA solution when confluence was reached and used in the experiments at the fourth passage.

### 2.3. Characterization of MSCs

Surface marker analysis of the isolated MSCs was performed by incubation with phycoerythrin-conjugated antibodies against murine CD11b, CD31, CD44, CD45, CD90.2, and Sca-1 (Invitrogen) for 30 min at 4°C. The cells were analyzed using a FACSAria III cytometer (Becton Dickinson, San Jose, CA) equipped with a 488 nm argon laser, and the graphics were generated in WinMDI 9.2 software. The adipogenic and osteogenic differentiation of the MSCs was performed according to protocols previously published [1]. After 4% paraformaldehyde fixation, the calcium deposition and lipid droplets were stained with Alizarin Red S and Oil Red O solution, respectively. The cell nuclei were stained with hematoxylin for adipogenic differentiation.

### 2.4. MSC Preactivation

The preactivation of the MSCs for the in vitro experiments and in vivo transplantation was performed by treating the cells with 20 ng/mL IFN-*γ* and 30 ng/mL TNF-*α* (PeproTech, Rocky Hill, NJ) for 20 h. Nonactivated cells, cultivated in standard culture medium, were used as the control group in almost all the experiments. To reduce the number of utilized animals, the islet transplantation experiment was made using only preactivated MCSs, due to their higher immunosuppressive potential, as shown in the in vitro assays.

### 2.5. Viability of Preactivated and Nonactivated MSCs

After preactivation, the MSCs were trypsinized and seeded in six-well dishes at 5 × 10^5^ cells per well. After 24 and 96 h, the MSCs were incubated with 50 *μ*g/mL propidium iodide in PBS for five minutes. Flow cytometry was used to determine the percentage of dead cells. The data was analyzed in BD FACSDiva 6.0 software.

### 2.6. MHC-II Expression in MSCs

After preactivation, the MSCs were trypsinized and seeded in six-well plates at 5 × 10^5^ cells per well. After 24 and 96 h, the MSCs were incubated with APC-conjugated anti-MHC class II (clone M5, Life Technologies) antibody for 30 min. The expression of MHC-II was analyzed by flow cytometry.

### 2.7. Splenocyte Proliferation Assay

BALB/c splenocytes were isolated by mechanical dissociation of the spleen followed by red blood cell lysis with 0.8% ammonium chloride solution. The splenocytes were plated at 10^5^ cells per well in 96-well plates. They were cocultivated in a 96-well plate with preactivated and nonactivated mitomycin-treated C57Bl/6- or BALB/c-derived MSCs in 200 *μ*L media of RPMI 1640, supplemented with 10% FBS, 0.1 mg/mL streptomycin, 100 U/mL penicillin, 55 *μ*M 2-mercaptoethanol, and 2 mM GlutaMAX Supplement (Gibco). Two MSC to splenocyte rates were used: 1 : 4 (2.5 × 10^4^ MSCs/well) and 1 : 10 (10^4^ MSCs/well). Three days after incubation, the cells were fixed and analyzed using a BrdU Cell Proliferation Kit (Millipore), according to the manufacturer's instructions. BrdU reagent was added to the culture medium 20 h prior to fixation. Sample absorbance was analyzed using a SpectraMax 190 Microplate Reader (Molecular Devices) at 450 nm wavelength.

### 2.8. Activation of CD4^+^ and CD8^+^ T Lymphocytes in Coculture with MSCs

BALB/c splenocytes were isolated, as previously described, and cocultured in a 12-well plate with preactivated or nonactivated C57Bl/6- or BALB/c-derived MSCs. Two MSC to splenocyte rates were used: 1 : 4 (1.25 × 10^5^ MSCs/well) and 1 : 10 (5 × 10^4^ MSCs/well). After 72 h, nonadherent cells were incubated with FITC-conjugated mouse CD4 and PE-conjugated mouse CD69 antibodies or FITC-conjugated mouse CD8 and PE-conjugated mouse CD69 antibodies, all from Invitrogen. Splenocytes were first gated using forward (FSC) and side scatter (SSC) properties. For this gate, additional gates on CD4^+^ or CD8^+^ populations were created. The percentage of cell activation was identified by the expression of CD69 in each population.

#### 2.8.1. Gene Expression of MSCs

The expression of inducible nitric oxide synthase (*Inos*), *CD274*, metalloproteinase 2 (*Mmp2*), cyclooxygenase 2 (*Cox2*), indoleamine (*Ido1*), and interleukin-6 (*Il6*) was quantified by RT-qPCR in IFN-*γ*-treated and IFN-*γ* plus TNF-*α*-treated mesenchymal stem cells after 20 h treatment. Nontreated MSCs were used as the control. The RNA was extracted using TRIzol reagent (Thermo Scientific) and resuspended in diethyl pyrocarbonate-treated water. Quantification and purity of the RNA were measured using a Nanodrop ND-2000, and the cDNA was synthesized using an M-MLV Reverse Transcriptase kit (Invitrogen). qPCR reactions were prepared using a SYBR® Green qPCR SuperMix kit (Invitrogen Co, Carlsbad, CA, USA). Each 20 *μ*L sample was composed of 10 *μ*L of qPCR Supermix, 500 nM ROX, 0.5 *μ*M of each primer (see Table S1 in Supplementary Material available online at https://doi.org/10.1155/2017/9824698), and 1 *μ*L of cDNA, after 1 : 3 dilution in water. qPCR cycle conditions were as follows: 95°C for 5 min for denaturation followed by 40 cycles at 95°C for 10 s, 60°C for 30 s, and the final step at 70°C for 10 min. All the samples were run in triplicate. Gene expression was normalized by *Actb* expression, and comparisons were performed using the untreated group as the reference, except for Ido, in which the group treated with IFN-*γ* was used. To calculate the relative expression of genes, the 2^−ΔΔCt^ method was used.

### 2.9. MSC Transplantation

Before MSC transplantation, the mice were divided into three groups. The syngeneic group (group 1) received 2 × 10^5^ GFP^+^ MSCs of C57Bl/6 mice transplanted into C57Bl/6 mice (*n* = 8). The allogeneic group (group 2) received 2 × 10^5^ GFP^+^ MSCs of C57Bl/6 GFP^+^ mice transplanted into BALB/c mice (*n* = 16). The allogeneic preactivated group (group 3) received 2 × 10^5^ preactivated GFP^+^ MSCs of C57Bl/6 mice transplanted into BALB/c mice (*n* = 16). After i.p. anesthesia, using 100 mg/kg ketamine and 10 mg/kg xilazine, the right kidney was exposed to receive the graft. Using a Hamilton syringe coupled to polyethylene tubing (PE50), the cells were transplanted into the subcapsular space of the kidney. The peritoneum and skin were closed with sutures. The animals received subcutaneous injections of 3 mg/kg butorphanol tartrate (Torbugesic®, Fort Dodge, USA) and were maintained on a heated pad until full recovery. The kidneys were recovered at 7, 14, 28, and 100 days after the transplant. After fixation with 10% formalin for 10 h, the kidneys were incubated with 30% sucrose solution in PBS for 24 h at 4°C. The cryopreserved tissue was embedded in freezing medium. Five- and ten-micron sections were collected using a cryostat.

### 2.10. Islet Isolation and Transplantation

Pancreatic islets were isolated, as previously described by Montaña et al. [27]. The pancreata were distended by the injection of 1 mg/mL collagenase type XI solution in RPMI 1640 followed by incubation at 37°C for 10 min. The tissue was then washed 3 times with RPMI 1640 supplemented with 10% bovine serum and penicillin/streptomycin and filtered in a strainer. The islets were isolated by centrifugation in Ficoll-Histopaque 1077 gradient at 10°C for 24 min, and 300 islets per animal were handpicked for transplantation under the kidney capsule. The islet alone or islet plus preactivated MSCs were pelleted by centrifugation in PE50 tubing and transplanted, as described for MSC transplantation.

### 2.11. Diabetes Induction and Blood Glucose Monitoring

Five days before transplantation, diabetes was induced in the mice by an i.p. injection of 180 mg/kg streptozocin (STZ) in citrate buffer, pH 4.0. The mice were considered diabetic and used as recipients when nonfasting blood glucose levels were above 360 mg/dL in two consecutive measurements. Five days after STZ induction, the mice were divided into three groups. The syngeneic group was composed of C57Bl/6 recipient mice, transplanted with 300 C57Bl/6 donor islets. The allogeneic group was composed of C57Bl/6 recipient mice, transplanted with 300 allogeneic BALB/c donor islets. The allogeneic plus MSC group was composed of C57Bl/6 recipient mice, transplanted with 300 allogeneic BALB/c donor islets and 2 × 10^5^ syngeneic preactivated MSCs. After transplantation, the blood glucose levels were measured daily from the tail vein using a glucometer (Accu-chek Performa, Roche). Graft rejection was defined as blood glucose > 280 mg/dL in at least three consecutive analyses. The mice were euthanized and the graft-bearing kidney was processed for histological analysis.

### 2.12. Histological Analysis and Immunofluorescence

The frozen kidney sections were stained with hematoxylin and eosin to evaluate the presence of MSCs and inflammatory cells at the transplant site. The sections were washed with PBS and incubated with PBS solution containing 1% BSA and 0.1% Triton X-100 for 30 minutes. Anti-GFP (A11122, Invitrogen) antibody was diluted to 1 : 500 and incubated with the sections for 1 hour at room temperature. After washing with PBS, incubation with Alexa Fluor® 568 goat anti-rabbit IgG diluted to 1 : 300 in PBS was performed. The cell nuclei were stained with 5 *μ*g/mL DAPI. The sections were analyzed using a Nikon inverted microscope.

### 2.13. Statistical Analysis

Statistical analysis was performed using SigmaPlot 12.0 software and the graphics were generated using GraphPad Prism 5. Statistical significance was evaluated using one-way and two-way ANOVA, followed by Tukey's post hoc test. A probability (*P*) value < 0.05 was considered significant.

## 3. Results

### 3.1. Characterization of MSCs

The analysis of surface markers on the MSCs at the fourth passage showed no expression of CD45, CD11b, and CD31 for more than 99% of the cells, which indicates there was no contamination with the hematopoietic cells. The cells expressed Sca-1 and especially CD44 and CD90.2 at high frequencies. The adipogenic and osteogenic differentiation assays showed that the cells readily differentiated along these lineages. In conclusion, the results of the characterization indicate that the cells used in this study were a purified population of MSCs (Figure S1).

### 3.2. Effects of MSC Preactivation

**Figure 1:**
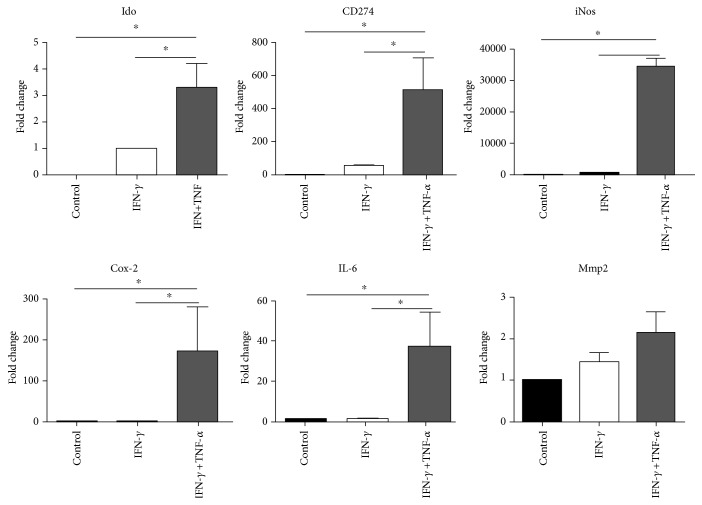
Increased expression of anti-inflammatory genes in preactivated mesenchymal stem cells. Gene expression analysis of murine MSCs was performed after preactivation with 20 ng/mL IFN-*γ* and 30 ng/mL TNF-*α* for 20 h. Expression changes on Il-6, Pd-l1 (CD274), iNos, Ido, Cox-2, and Mmp2 were analyzed by qPCR on preactivated cells and compared to the control group (untreated cells). Data were expressed as fold change ± SD of three independent experiments.^∗^*P* < 0.05. One-way ANOVA, post hoc Tukey.

Assays were performed to evaluate the effect of the preactivation protocol on gene expression, cellular viability, MHC class II expression, splenocyte proliferation, and T cell activation. Changes in the expression of immune-related genes were analyzed on the MSCs treated with IFN-*γ* alone and IFN-*γ* plus TNF-*α*. The untreated MSCs expressed all the cytokines evaluated except for *Ido1*. The expression of *Ido1* slightly increased 3.3 times in INF-*γ* + TNF-*α*-treated cells compared to the IFN-*γ*-treated cells (Figure 1). *CD274* and *iNos* expression showed approximately 513- and 34,451-fold increase, respectively (Figure 1), on the IFN-*γ* + TNF-*α*-treated cells but were not altered by the IFN-*γ* alone. Similarly, *Il-6* and *Cox-2* gene expression was significantly upregulated by 173- and 38-fold, respectively, in response to IFN-*γ* + TNF-*α*, but no significant changes were observed in the IFN-*γ*-only treated group. No changes in *Mmp-2* expression were detected (Figure 1).

**Figure 2:**
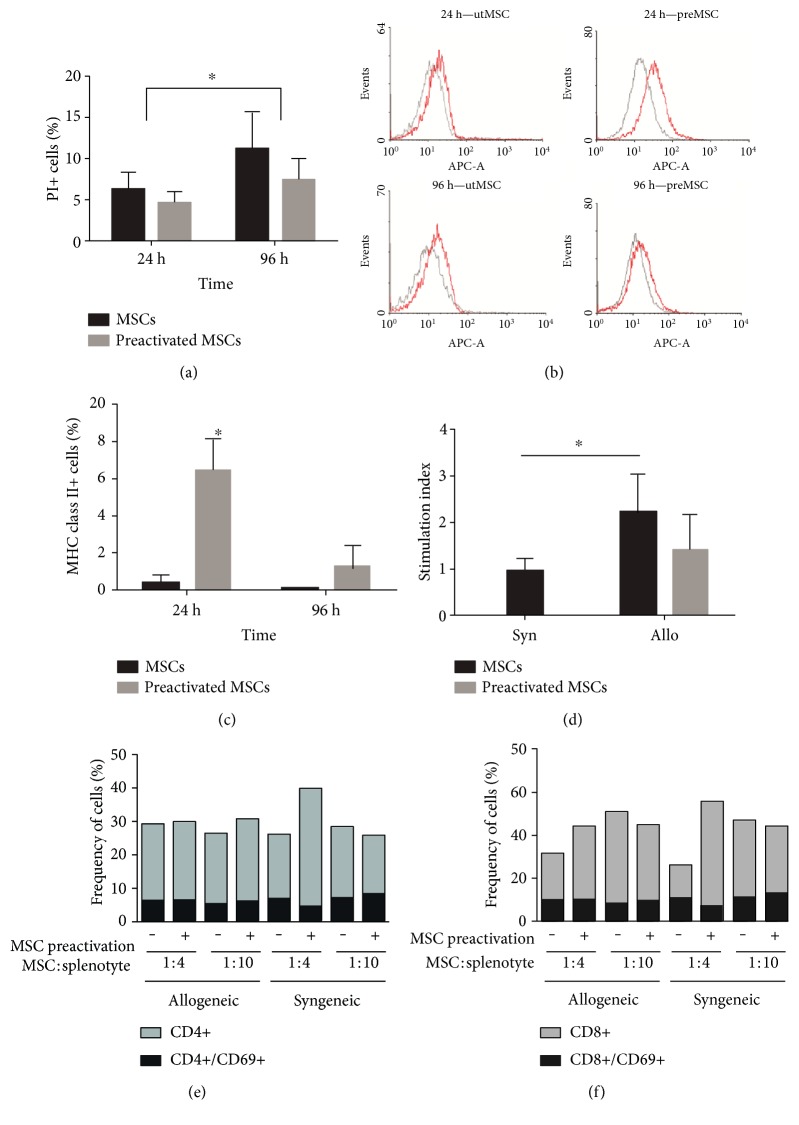
Cellular characterization after preactivation of MSCs. Viability test and MHC class II expression were performed 24 h and 96 h after MSC preactivation with 20 ng/mL IFN-*γ* and 30 ng/mL TNF-*α* for 20 h. The frequency of dead cells (a) in the preactivated and untreated groups was estimated by propidium iodide incubation and flow cytometry analysis (*n* = 3). MHC-II expression (b) was assessed by anti-MHC class II antibody (red peaks) and isotype control (grey peaks) on the preactivated and untreated MSCs after 24 and 96 h. The frequency of MHC class II-positive cells (c) from three independent experiments is presented (*n* = 3). Splenocyte proliferation assay was performed by coculture of splenocytes with preactivated and untreated allogeneic or syngeneic MSCs. Proliferation was measured by BrdU incorporation after 72 h coculture with the preactivated and untreated MSCs (d). The stimulation index (SI) was calculated by the ratio between the absorbance of the MSC/splenocyte coculture group and the splenocytes only (control group). Analysis of activated T cells after coculture for 72 hours with syngeneic or allogeneic mesenchymal stem cells (e and f). No statistically significant differences were found in the frequency of activated T helper cells (CD4^+^/CD69^+^) (e) or T cytotoxic cells (CD8^+^/CD69^+^) (f) when splenocytes were cocultured with syngeneic or allogeneic MSCs at 1 : 4 or 1 : 10 MSC to splenocyte rates (*n* = 3). Two-way ANOVA, post hoc Tukey. Allo: allogeneic MSCs; Syn: syngeneic MSCs. Significant difference between allogeneic (*n* = 5) and syngeneic (*n* = 3) groups. Two-way ANOVA, post hoc Tukey. ^∗^*P* < 0.05.

The viability test was performed to evaluate any harmful effect of preactivation. The results showed no statistical difference in the number of dead cells in the preactivated MSCs in comparison with the untreated group after 24 and 96 h post treatment (Figure 2(a)). Therefore, the preactivation of MSCs does not cause a decrease in cellular viability (Figure 2(a)). The proinflammatory effect of IFN-*γ* plus TNF-*α* treatment has the potential of increasing MHC class II expression. Flow cytometry analysis showed a low frequency of MHC class II-expressing cells on the untreated MSCs (Figures 2(b) and 2(c)). Preactivation significantly enhanced overall MHC class II molecules on the surface of the MSCs (Figure 2(b)), increasing the frequency of positive cells to approximately 6% after 24 h. However, this increase was transient and returned to values similar to the untreated cells after 96 h (Figures 2(b) and 2(c)). The mixed MSC : splenocyte coculture stimulated splenocyte proliferation in a direct contact configuration. This effect was diminished by the previous preactivation of the MSCs but was still higher than that of the control group (Figure 2(d)). Analysis of T cell activation by the syngeneic and allogeneic MSCs was evaluated by the number of CD4^+^/CD69^+^ and CD8^+^/CD69^+^ T cells. The frequency of activated cells showed no significant difference in the coculture with the untreated or preactivated MSCs after 72 h incubation. Both the syngeneic and allogeneic MSCs had similar proportions of activated CD4^+^ (Figure 2(e)) and CD8^+^ T (Figure 2(f)) lymphocytes. The MSC to lymphocyte ratio in culture made no difference to the CD69^+^ frequency (Figures 2(e) and 2(f)).

### 3.3. Permanence of MSCs In Vivo

**Figure 3:**
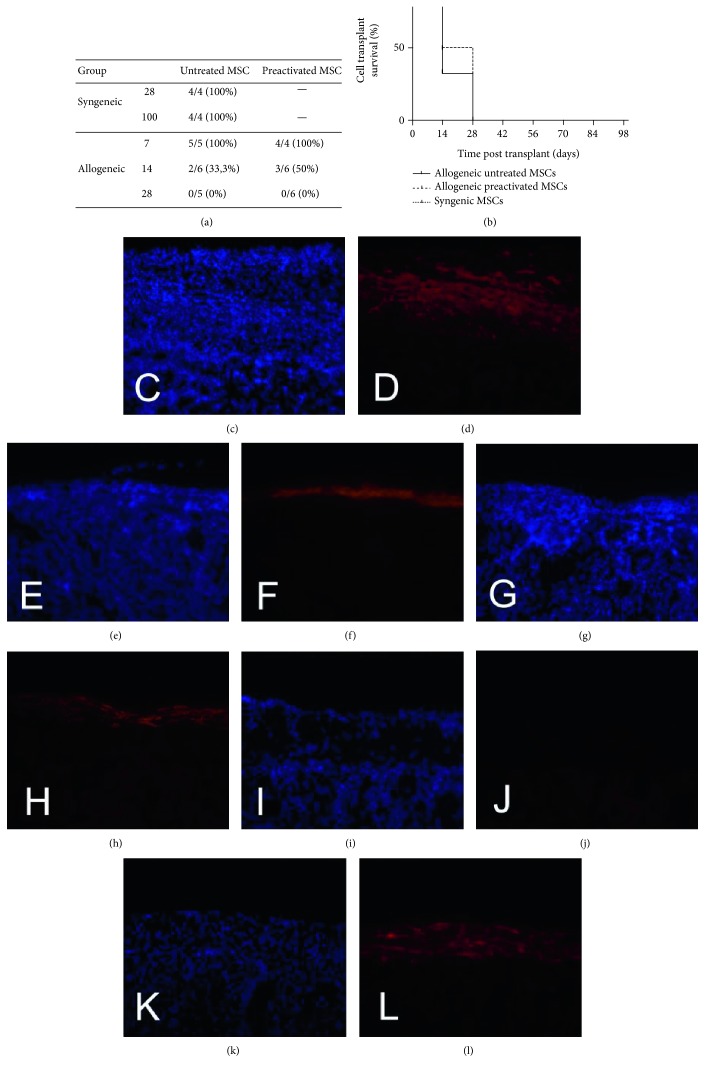
Analysis of graft-bearing kidneys for the presence of GFP^+^ transplanted cells. After immunofluorescence analysis of the graft-bearing kidneys using anti-GFP antibody, the number of mice with remaining GFP^+^ cells at the transplant site and the number of mice analyzed in each group were tabulated (a). No differences in the frequency of graft survival between the untreated and preactivated allogeneic groups were observed (b); differences were only found between the allogeneic and syngeneic groups, *P* < 0.01, log rank. The allogeneic group 7 days after transplantation (c, d), stained with DAPI and GFP^+^, respectively. The allogeneic group 14 days after transplantation (e, f). The allogeneic group 14 days after transplantation of preactivated MSCs (g, h). The allogeneic group 28 days after transplantation (i, j). The syngeneic group 100 days after transplantation (k, l). Magnification of 200x.

**Figure 4:**
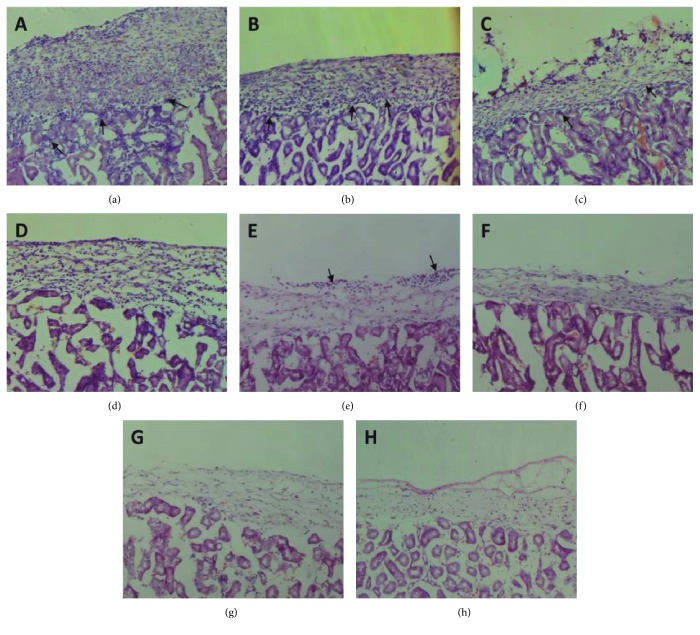
Lymphocytic infiltration analysis of the graft-bearing kidneys at different time periods stained with hematoxylin and eosin. Allogeneic MSC and allogeneic + preactivated MSCs (b) after 7 days post transplantation. Allogeneic MSC (c) and allogeneic + preactivated MSCs (d) after 14 days post transplantation. Allogeneic MSC (e) and allogeneic + preactivated MSCs (f) 28 days post transplantation. Syngeneic MSC (g) 28 days post transplantation and syngeneic MSC (h) 100 days post transplantation. Magnification of 100x.

The survival of syngeneic and allogeneic MSCs was tested. Grafts were analyzed after 7, 14, and 28 days posttransplantation for the allogeneic MSCs and 28 and 100 days for the syngeneic MSCs (Figure 3(a)). GFP^+^ cells were observed at the transplant site of all the syngeneic-transplanted mice at 28 and 100 days (Figure 3(l)). The presence of both the untreated and preactivated allogeneic MSCs was observed at day 7. At 14 days, 2 out of 6 mice (33.3%) transplanted with untreated MSCs (Figure 3(f)) did not present GFP^+^ cells at the transplant site compared to 3 out of 6 (50%) that received preactivated MSCs (Figure 3(h)). No GFP^+^ cells were found in the allogeneic groups after 28 days (Figures 3(b) and 3(j)). As shown in Figure 3(b), the preactivation did not change the allogeneic graft endurance time. The only difference found was between the allogeneic and syngeneic graft survival time (Figure 3(b), *P* < 0.01). The mice transplanted with untreated (Figure 4(a) and preactivated (Figure 4(b)) allogeneic MSCs after 7 and 14 days (Figures 4(c) and 4(d)) exhibited infiltrated immune cells at the transplant site. Little or no infiltration of inflammatory cells was found after 28 days of the untreated (Figure 4(e)) and preactivated MSCs (Figure 4(f)). No inflammatory infiltration was observed in the syngeneic-transplanted group after 28 (Figure 4(g)) and 100 days (Figure 4(h)).

### 3.4. Preactivated MSCs Prolong Allogeneic Islet Transplantation

**Figure 5:**
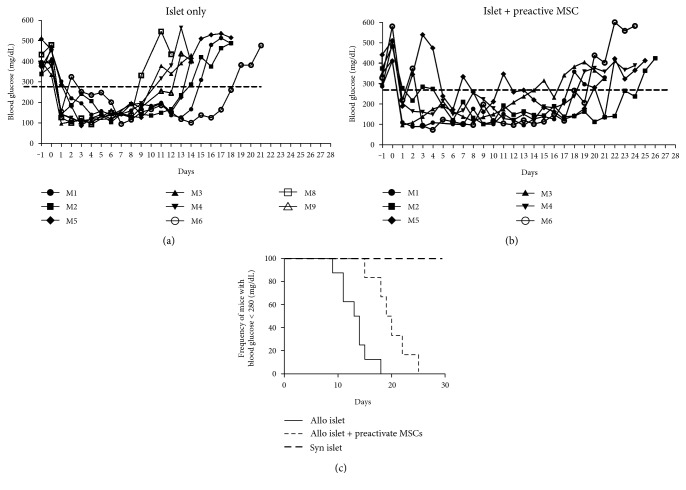
Cotransplantation of preactivated MSCs prolongs islet graft survival in mice. Blood glucose concentration was measured daily in the mice transplanted with 300 islets alone (a) (*n* = 8) or 300 islets + 2 × 10^5^ preactivated MSCs (b) (*n* = 6). Graft rejection was confirmed after two consecutive blood glucose measurements above 280 mg/dL. The percentage of normoglycemic mice overtime was compared between the groups (c). Average rejection time of the islet alone group was 13.12 ± 2.80 days compared to 19.83 ± 3.43 in the islet + preactivated MSCs. The mice transplanted with syngeneic islet (*n* = 3) remained normoglycemic for at least 30 days after transplantation. *P* < 0.0021, log-rank test.

As preactivation of the MSCs increased the expression of the anti-inflammatory molecules and increased in vitro immunosuppressive properties, we decided to test the role of the preactivated MSCs on the allogeneic islet transplantation outcome. The results show that in both groups, the transplanted mice restored blood glucose levels after transplantation (Figure 5). The mice transplanted with islet alone (Figure 5(a)) took an average of 13.1 ± 2.8 days to become diabetic again, compared to 19.8 ± 3.4 days with the islet plus preactivated MSCs (Figure 5(b)) (*P* < 0.0021, log-rank test (Figure 5(c)). It suggests that preactivated MSCs enhance allograft survival of islets transplanted in diabetic mice.

## 4. Discussion

Strategies to prolong allogeneic MSC engraftment in vivo have been reported [28–34]. The most common approach to MSC modification relates to the genetic manipulation by superexpressing or blocking genes involved in the immune recognition [29–31]. However, this approach requires complex techniques and presents safety issues that hinder translation to the clinics [30, 31]. For these reasons, cytokine modulation of cells is a more feasible and less complex approach [32]. As previously described in the literature, the immunomodulatory potential of MSCs could be altered by IFN-*γ* treatment, potentiating their immunosuppressive characteristics [20, 24, 33, 34]. The present results show a significant upregulation in *iNos*, *CD274*, *Cox-2*, *Ido1*, and *il6* expression after cytokine preactivation, especially if using IFN-*γ* along with TNF-*α*. Nitric oxide produced by the activity of iNos abolishes T cell proliferation [35]. Similarly, CD274 plays a critical role in immunosuppression through binding with the PD-1 receptor, which results in the inactivation of B and T cells [33, 36]. By acting through tryptophan depletion, Ido is described to be the major molecule involved in immunosuppression of human MSCs [5, 10]. The low expression of MHC-II on MSCs is already described in the literature [37]. The present results show a slight increase in MHC-II expression after 20 h preactivation that could potentially raise their in vivo allorecognition.

Unlike the previous in vitro studies [4, 5, 37], the splenocytes and allogeneic MSC cocultures stimulated in vitro proliferation of splenocytes, indicating MSC allorecognition. The preactivation of MSCs leads to a significant reduction of splenocyte proliferation in the allogeneic cocultures, probably by upregulation of anti-inflammatory molecules [20]. As demonstrated in the previous studies, the MSCs had the ability of suppressing T lymphocyte proliferation in vitro [4, 37]. This contrast may be attributed to the use of mitogens to activate T lymphocytes, which alter local inflammatory microenvironment, leading to changes in the secretion pattern of MSCs. Without these stimuli, upregulation of anti-inflammatory molecules could be delayed, leading to higher proliferation of T lymphocytes, especially in the early stages. The frequencies of activated CD4^+^/CD69^+^ and CD8^+^/CD69^+^ T cells were similar in the syngeneic and allogeneic cocultures. In a similar study, the suppression of T lymphocyte proliferation in a coculture with MSCs was not related to the reduction of CD25^+^ or CD69^+^ T lymphocytes [34] and can be attributed to the effect of nitric oxide on T cells, which inhibits mitosis, even on activated T cells [38]. This data could indicate why preactivated allogeneic MSCs tend to be less stimulatory than nonactivated allogeneic cells, even though they present an increase in MHC-II expression.

In vivo, allogeneic MSC rejection occurred 7 and 28 days after transplantation. At 14 days, some but not all mice still presented allogeneic MSCs at the transplant site. The rejection of allogeneic MSCs occurred later than the other allogeneic cell types, such as fibroblasts, which endured for about 10 days [39]. Another study reported allogeneic MSC rejection between 20–40 days and fibroblasts less than 20 days after intraperitoneal or intravenous transplant [18]. In a similar result, both syngeneic and allogeneic bone marrow-derived MSCs showed a significant decrease in the number of transplanted cells from day 7 to day 14 in a model of skin regeneration and very few cells were observed at day 28 [40]. On the other hand, studies have shown survival reduction of allogeneic MSCs, in comparison with autologous MSCs, in mice [14, 39]. Allogeneic MSCs can persist longer within an immune-suppressed environments, such as tumors, or immune-privileged sites. It indicates that local immune suppression could mask MSC immunogenicity [41]. Camp et al. demonstrated the presence of allogeneic MSCs 40 days after intracranial transplantation in rats [18]. Other factors, such as MSC heterogeneity, donor, source, passage number, and even culture conditions, can lead to different MSC responses in similar contexts. A study demonstrated that high-passage MSCs provoked a larger inflammatory reaction than low-passage cultures after systemic infusion in patients with GVHD [42].

The present results corroborate findings from other studies that showed that allogeneic MSCs can be recognized and that they evoked an immune cell response in vivo. This might have occurred due to the inflammatory context encountered by the cells after transplantation, which could modulate MHC class I and II expression and increase their immunogenic potential. Data shows that about 13% of patients that received allogeneic MSCs presented antibodies against the donor [43] and repeated doses are frequently administrated without any harmful complications [8]. The consequences of this for the clinical application of allogeneic expanded cells are not clear. Studies suggest that the therapeutic effect of MSCs is not dependable on their persistence at the transplant site [44]. The factors secreted by the cells in the first days after transplantation seem to be responsible for their therapeutic properties, and this was named the hit-and-run effect of MSCs [44]. This characteristic could be extremely helpful to control acute inflammatory processes and control immune-related diseases, even without long-term persistence on the host. However, the use of allogeneic MSCs with the aim of replenishing cells or tissue should be reconsidered due to their proven allorecognition.

The cotransplantation of islets with MSCs has been reported [45–48]. Allogeneic islet rejection was prevented for more than 90 days after cotransplantation with an MSC [45]. Two other studies showed prolonged islet survival after intravenous MSC injections [46] or transplantation under the kidney capsule [49]. The mice become hyperglycemic from day 8 to 16 in the islet alone group, while in the MSC-coinjected group, a more gradual (day 12 to 28) and slow increase in glucose levels occurred. The MSC effect was enhanced by associated anti-CD45RB immunotherapy [46].

A delayed rejection time, as well as decreased lymphocytic infiltration and autoantibody levels, were also observed in nonobese diabetic mice [47]. In contrast, no effect on allogeneic islet survival was reported by using untreated MSCs in the rats, except when associated with subtherapeutic doses of cyclosporine A, which increased the graft survival time to 89.3 days compared to 7.8 days for the islet alone group [48]. A great variability in the capacity of MSCs in preventing islet rejection was observed. No studies employing preactivated MSCs on islet transplantation were found, making comparison difficult. The survival of allogeneic islets cotransplanted with syngeneic MSCs after preactivation was prolonged for about 6 days, compared with that of the islet alone group. Further studies are required to clarify the role of untreated and preactivated MSCs on allogeneic islet transplantation outcome.

In conclusion, it has been demonstrated here that murine allogeneic MSCs can elicit an immunogenic response when transplanted into an immune-competent host, leading to full graft rejection. Preactivation of allogeneic MSCs did not prevent rejection but enhanced their immunomodulatory capacity in vitro. In contrast, preactivated syngeneic MSCs prolong allogeneic islet survival and present promising potential for use in clinical studies for allogeneic transplantation. New strategies to reduce the immunogenicity of allogeneic MSCs must be tested to increase their in vivo permanence and therapeutic effects on organ transplantation.

## Supplementary Material

Table S1. Primer sequences and amplicon characteristics. Figure S1. Characterization of adipose-derived mesenchymal stem cells at passage 5. (A) The cells were immunophenotyped for the expression of CD11b, CD31, CD44, CD45, CD90.2 and Sca-1 by flow cytometry. At least 5.000 events were analyzed. (B) Cell morphology was analyzed by phase contrast (B) and fluorescence microscopy (C). Adipogenic and osteogenic differentiation of adipose-derived mesenchymal stem cells at passage 5. MSCs cultured for 4 weeks in adipogenic (D) and osteogenic (F) media and respective control group (E, G). Cells stained with Oil Red O (D, E) and (F, G) Alizarin Red S. Magnification 100X.

## Abbreviations

APCs: Antigen-presenting cells
Cox-2: Cyclooxygenase 2
i.p.: Intraperitoneal
Ido: Indoleamine
Il-6: Interleukin 6
iNos: Inducible nitric oxide synthase
Mmp2: Matrix metalloproteinase 2
MSCs: Mesenchymal stem cells
Pd-l1: Programmed death-ligand 1
STZ: Streptozotocin.
