# Supplementary material for: In Vivo Immunogenic Response to Allogeneic Mesenchymal Stem Cells and the Role of Preactivated Mesenchymal Stem Cells Cotransplanted with Allogeneic Islets
Source: Stem Cells Int. 2017 May 3;2017:9824698. doi: 10.1155/2017/9824698 (PMC5434460; doi:10.1155/2017/9824698)

***in vivo* immunogenic response to allogeneic mesenchymal stem cellS and the role of preactivated mesenchymal stem cellS CO-TRANSPLANTED with allogeneic islets**

Régis Linhares Oliveira ^1,3^; Pedro Cesar Chagastelles ^1,3^; Patrícia Sesterheim ^4^; Patricia Pranke ^1,3,5^

^1^Hematology and Stem Cell Laboratory, Faculty of Pharmacy, Universidade Federal Rio Grande do Sul; ^2^ Stem Cell Laboratory, Fundamental Health Science Institute, Universidade Federal Rio Grande do Sul; ^3^ Post Graduate Program in Physiology, Universidade Federal Rio Grande do Sul; ^4^ Fundação Estadual de Produção e Pesquisa em Saúde; ^5^Stem Cell Research Institute; Brazil

**Supplementary Subtitles of Figures and Tables**

Table S1. Primer sequences and amplicon characteristics.

Figure S1. Characterization of adipose-derived mesenchymal stem cells at passage 5. (A) The cells were immunophenotyped for the expression of CD11b, CD31, CD44, CD45, CD90.2 and Sca-1 by flow cytometry. At least 5.000 events were analyzed. (B) Cell morphology was analyzed by phase contrast (B) and fluorescence microscopy (C). Adipogenic and osteogenic differentiation of adipose-derived mesenchymal stem cells at passage 5. MSCs cultured for 4 weeks in adipogenic (D) and osteogenic (F) media and respective control group (E, G). Cells stained with Oil Red O (D, E) and (F, G) Alizarin Red S. Magnification 100X.

**Supplementary Figures and Tables**

Table S1.
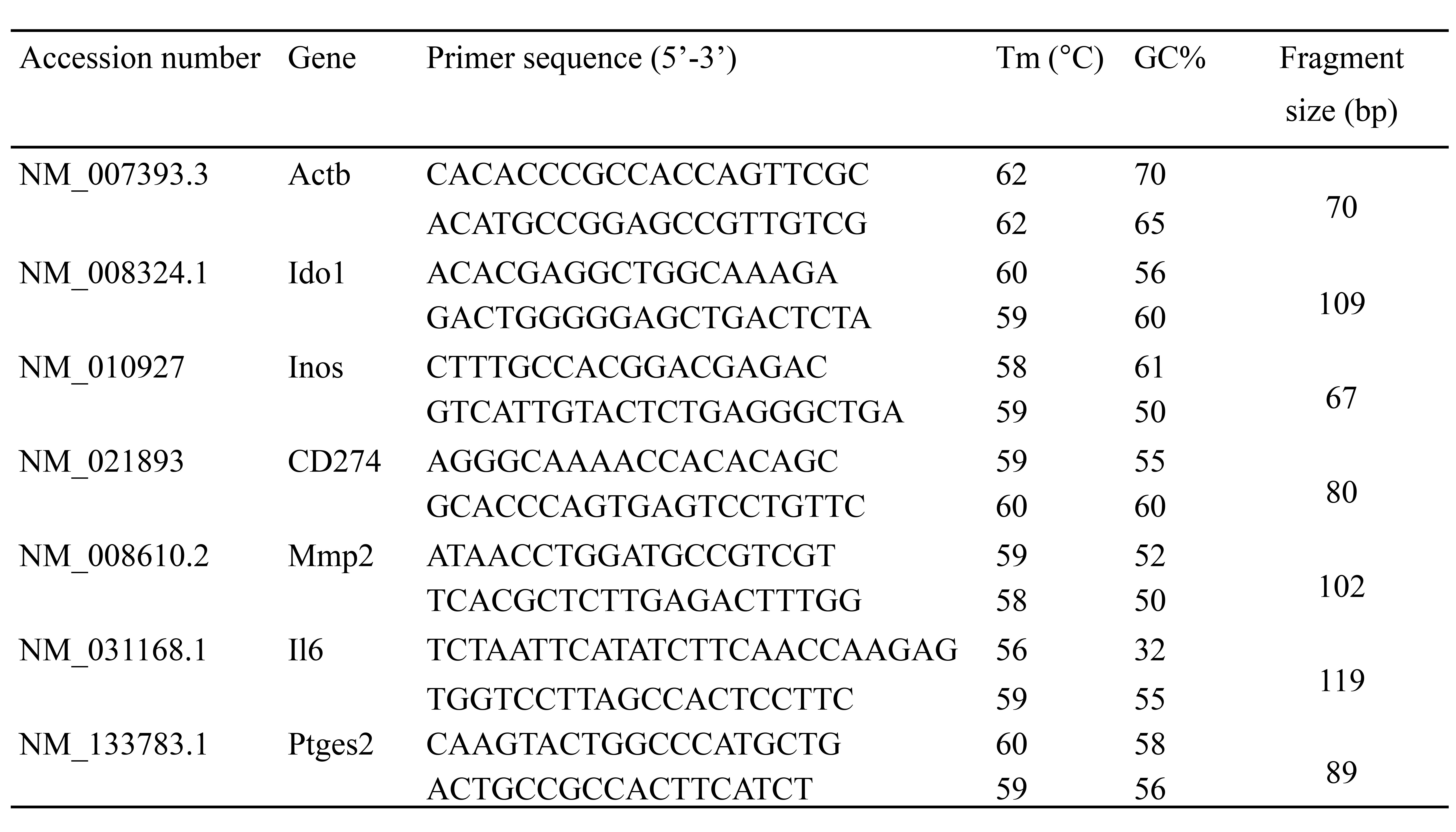


Figure S1


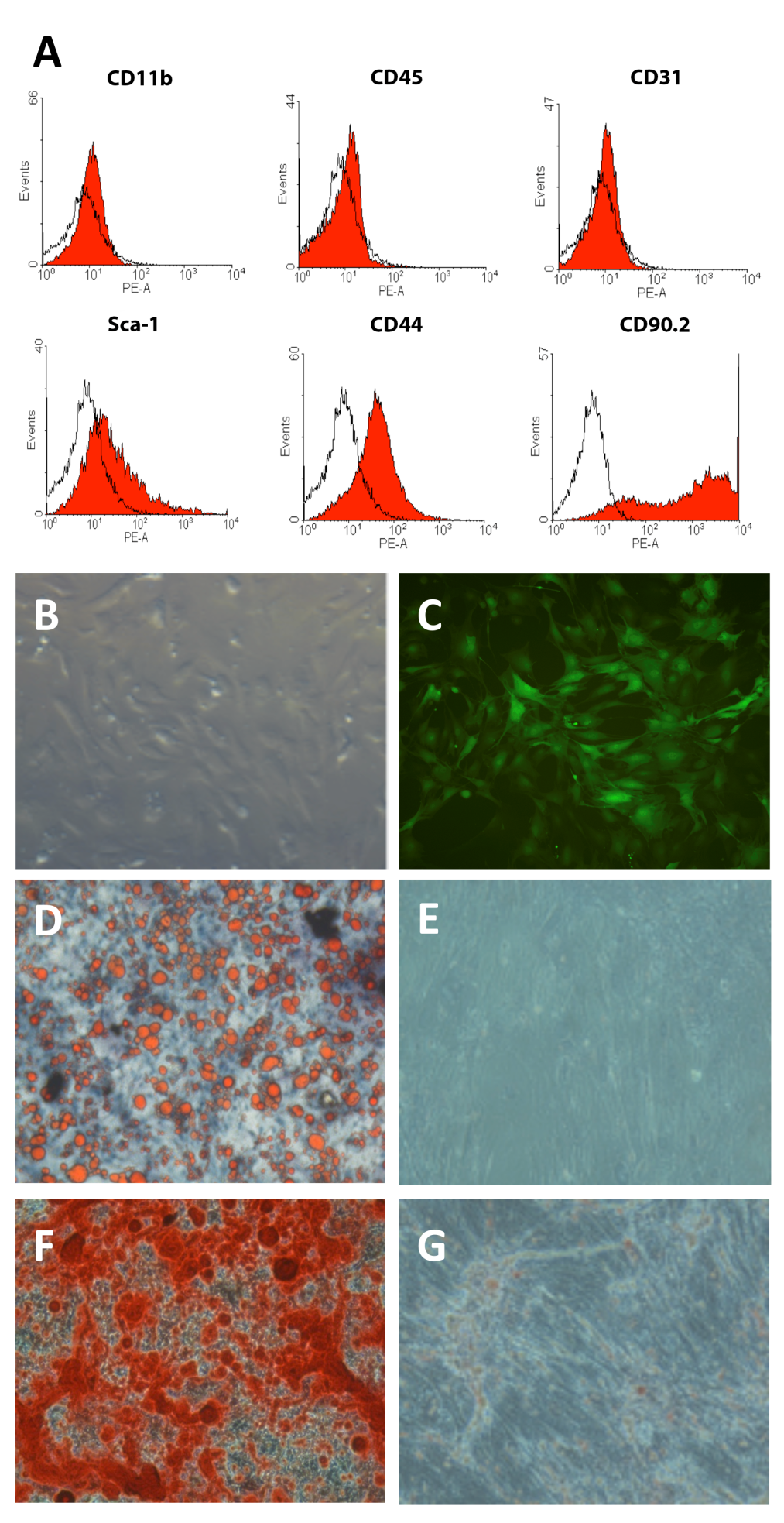

Supplement: Supplementary file 1 — Table S1. Primer sequences and amplicon characteristics. Figure S1. Characterization of adipose-derived mesenchymal stem cells at passage 5. (A) The cells were immunophenotyped for the expression of CD11b, CD31, CD44, CD45, CD90.2 and Sca-1 by flow cytometry. At least 5.000 events were analyzed. (B) Cell morphology was analyzed by phase contrast (B) and fluorescence microscopy (C). Adipogenic and osteogenic differentiation of adipose-derived mesenchymal stem cells at passage 5. MSCs cultured for 4 weeks in adipogenic (D) and osteogenic (F) media and respective control group (E, G). Cells stained with Oil Red O (D, E) and (F, G) Alizarin Red S. Magnification 100X. [file 9824698.f1.docx]
